# A novel 7-chemokine-genes predictive signature for prognosis and therapeutic response in renal clear cell carcinoma

**DOI:** 10.3389/fphar.2023.1120562

**Published:** 2023-03-20

**Authors:** Ming-Jie Lin, Xiu-Xiao Tang, Gao-Sheng Yao, Zhi-Ping Tan, Lei Dai, Ying-Han Wang, Jiang-Quan Zhu, Quan-Hui Xu, Mukhtar Adan Mumin, Hui Liang, Zhu Wang, Qiong Deng, Jun-Hang Luo, Jin-Huan Wei, Jia-Zheng Cao

**Affiliations:** ^1^ Department of Urology, The First Affiliated Hospital of Sun Yat-sen University, Guangzhou, China; ^2^ Advanced Medical Technology Center, The First Affiliated Hospital, Zhongshan School of Medicine, Sun Yat-sen University, Guangzhou, China; ^3^ Department of Urology, Affiliated Longhua People’s Hospital, Southern Medical University, Shenzhen, China; ^4^ Institute of Precision Medicine, The First Affiliated Hospital of Sun Yat-sen University, Guangzhou, China; ^5^ Department of Urology, Jiangmen Central Hospital, Jiangmen, Guangdong, China

**Keywords:** chemokine, renal clear cell carcinoma, immunotherapy, tumor microenvironment, gene signature

## Abstract

**Background:** Renal clear cell carcinoma (ccRCC) is one of the most prevailing type of malignancies, which is affected by chemokines. Chemokines can form a local network to regulate the movement of immune cells and are essential for tumor proliferation and metastasis as well as for the interaction between tumor cells and mesenchymal cells. Establishing a chemokine genes signature to assess prognosis and therapy responsiveness in ccRCC is the goal of this effort.

**Methods:** mRNA sequencing data and clinicopathological data on 526 individuals with ccRCC were gathered from the The Cancer Genome Atlas database for this investigation (263 training group samples and 263 validation group samples). Utilizing the LASSO algorithm in conjunction with univariate Cox analysis, the gene signature was constructed. The Gene Expression Omnibus (GEO) database provided the single cell RNA sequencing (scRNA-seq) data, and the R package “Seurat” was applied to analyze the scRNA-seq data. In addition, the enrichment scores of 28 immune cells in the tumor microenvironment (TME) were calculated using the “ssGSEA” algorithm. In order to develop possible medications for patients with high-risk ccRCC, the “pRRophetic” package is employed.

**Results:** High-risk patients had lower overall survival in this model for predicting prognosis, which was supported by the validation cohort. In both cohorts, it served as an independent prognostic factor. Annotation of the predicted signature’s biological function revealed that it was correlated with immune-related pathways, and the riskscore was positively correlated with immune cell infiltration and several immune checkpoints (ICs), including CD47, PDCD1, TIGIT, and LAG-3, while it was negatively correlated with TNFRSF14. The CXCL2, CXCL12, and CX3CL1 genes of this signature were shown to be significantly expressed in monocytes and cancer cells, according to scRNA-seq analysis. Furthermore, the high expression of CD47 in cancer cells suggested us that this could be a promising immune checkpoint. For patients who had high riskscore, we predicted 12 potential medications.

**Conclusion:** Overall, our findings show that a putative 7-chemokine-gene signature might predict a patient’s prognosis for ccRCC and reflect the disease’s complicated immunological environment. Additionally, it offers suggestions on how to treat ccRCC using precision treatment and focused risk assessment.

## 1 Introduction

Kidney cancer is among the top 10 most common cancers, with 400,000 new cases and 175,000 deaths from cancer globally each year, and it accounts for 4% of all newly diagnosed cancers. (Kotecha et al., 2019; Siegel et al., 2021). Renal clear cell carcinoma (ccRCC) is the most prevalent subtype of the disease and is one of the principal reasons for patient death. (Hsieh et al., 2017). Surgery remains the primary therapy for kidney cancer patients, and despite the popularity of some emerging treatments, many patients develop distant metastases and locally advanced disease. (Capitanio and Montorsi, 2016). Therefore, novel treatment strategies are urgently needed.

Immunotherapy is a powerful treatment approach that has changed the landscape of treatment for many tumors. (Riley et al., 2019; Kennedy and Salama, 2020). Immunotherapy remains a promising therapeutic approach in the field of kidney cancer, and more new and rational immunotherapy approaches are needed besides targeting PD-1, CTLA4 or PD- L1. (Braun et al., 2021). The successful realization of immunotherapy cannot be achieved without the contribution of the TME. Chemokines have been found to either directly or indirectly affect tumor immune in the TME. (Nagarsheth et al., 2017). It is noteworthy that chemokines can entice various immune cells to reach the TME.

Chemokines are divided into four families: CC-chemokines, CXC-chemokines, XC-chemokines and CX3C-chemokines. (Griffith et al., 2014). They are not only involved in tumor proliferation and invasion, but also in inflammatory response and regulation of neoangiogenesis. (Ozga et al., 2021). The chemokine system is intricate, as shown by the fact that a single chemokine can draw in and activate both pro- and anti-tumor regulatory cells, hence promoting both pro- and anti-tumor actions. (Ozga et al., 2021). Studies have been performed to analyze the tertiary lymphoid structure-related chemokines in ccRCC. (Xu et al., 2022). In glioma and lung squamous cell carcinoma, comprehensive analyze of chemokines have been performed through public transcriptome databases, (Fan et al., 2022; Lai et al., 2022), while in ccRCC there are rarely. Thus, there is a need to further analyze the relationship between chemokines and ccRCC.

There is increasing evidence that chemokines are involved in the pathophysiological processes of tumors. (Reschke and Gajewski, 2022). Thus, it is necessary to incorporate chemokines into preclinical models to develop prognostic signature and new therapeutic targets. To address the above issues, we sought to apply chemokine family genes to develop and validate risk stratification signature of ccRCC patients from an independent public database to assess prognosis and discover new candidate drugs. This work may help to optimize precise treatment and further improve clinical outcomes for patients with ccRCC.

## 2 Materials and methods

### 2.1 Acquisition of samples and datasets

We downloaded clinicopathological information and RNA sequencing data for ccRCC patients from The Cancer Genome Atlas (TCGA) database (accessed on 2022/9/11 at https://xenabrowser.net/datapages/). Transcripts per kilobase million (TPM) values were derived by converting the gene expression values (FPKM values) from the RNA sequencing data. This study used 526 ccRCC tumor samples, which were randomly split into a training cohort (*N* = 263) and a validation cohort (*N* = 263) in a 1:1 ratio. All 526 qualifying ccRCC patients’ clinical features were compiled (SupplementaryTable S1). Patients whose survival information was unknown were excluded from further analysis.

### 2.2 Construction of 7-chemokine-genes signature

Firstly, we conducted differential expression analysis on training cohort samples and normal samples using the R package “DESeq2” to find differentially expressed genes (DEGs). According to the criteria of | $\log_{2}$ FoldChange|>1 & *p* < 0.05, we deemed them statistically significant. The 37 chemokine-related genes we chose to focus on were then intersected with these genes. After taking the intersection, genes associated with prognosis were further filtered using univariate Cox regression analysis. Lastly, seven genes were identified using the least absolute shrinkage and selection operator machine learning algorithm (LASSO). (Tibshirani, 1997). The expression values of the seven chemokine genes were multiplied by their corresponding correlation coefficients and then summed to obtain the riskscore.

### 2.3 Validation of 7-chemokine-genes signature

The median riskscore used as the dividing line between the high-risk and low-risk patient groups. In order to evaluate the effectiveness of the signature’s predictive ability, we conducted survival analysis, receiver operating characteristic (ROC) curve, univariate, and multivariate analysis. The “survival” package was used to run survival analysis on the training and validation cohorts. ROC curves for 1, 3, and 5 years were plotted using the “timeROC” R package. Using IBM SPSS Statistics 26, univariate and multivariate Cox regression analysis were carried out.

### 2.4 Construction of a nomogram for evaluation of gene signatures

Nomogram analysis was carried out for the training and validation cohorts using the R package “rms”. The scoring system and prediction system are located in the upper and bottom portions, respectively. In ccRCC patients, the overall score and the sum of the scores for each component correctly predicted the 1-, 3-, and 5-year survival. For both cohorts of patients, the predictive accuracy of OS was validated. To demonstrate the accuracy of survival prediction, calibration curves and C-index values were used.

### 2.5 GO analysis and gene set variation analysis (GSVA)

By using Pearson correlation analysis (R > 0.3, *p* < 0.05), it was possible to identify genes that were positively correlated with riskscore. These genes were then uploaded to the DAVID database (https://david.ncifcrf.gov/home.jsp) for annotation, visualization, and integrated discovery. *Homo sapiens* was chosen as the species and official gene symbol as identifier. The Gene Ontology (GO) study produced rich results in the end. The MSigDB database was used to acquire the HALLMARK gene set. Using the “GSVA” package (Hänzelmann et al., 2013), functional enrichment scores were computed for each sample, and a heatmap of the data was created (https://www.xiantao.love/). To ascertain the relationship between the riskscore and the HALLMARK set, Pearson correlation analysis was used.

### 2.6 Gene mutation analysis

The TCGA database (https://portal.gdc.cancer.gov/) was utilized to get somatic mutation data, which were analyzed using the “maftools” R package. The tumor mutation burden (TMB), based on somatic mutation data, was then determined for each patient in the training cohort, and the TMB between the high-risk and low-risk groups was compared. The TMB score was used as the basis for the survival analysis.

### 2.7 scRNA-seq analysis

The Gene Expression Omnibus (GEO) database provided the scRNA-seq data GSE152938 for ccRCC. Seurat is a R package for scRNA-seq expression data quality control, normalization, downscaling, and processing. It was used to evaluate the expression of 7 chemokine genes in tumor tissues. Expression data were normalized and downscaled for clustering using the UMAP method. Cellular markers were obtained from “CellMarker” (http://xteam.xbio.top/CellMarker/).

### 2.8 Evaluation of immune cell infiltration status

We evaluated the absolute proportion of 22 infiltrating immune cells in the training cohort ccRCC samples using the “CIBERSORT” algorithm in order to investigate the relationship between riskscore and immune cell infiltration. (Newman et al., 2015). The relative abundance of each TME cell infiltration in the training cohort ccRCC samples was also determined using the single sample gene set enrichment analysis (ssGSEA) algorithm. (Hänzelmann et al., 2013).

### 2.9 Drug sensitivity analysis

An R package called “pRRophetic” can analyze gene expression data to forecast the effectiveness of clinical chemotherapy and the sensitivity to targeted treatments. (Geeleher et al., 2014). Based on gene expression and drug sensitivity data from Cancer Genome Project (CGP) cell lines, we utilize the “pRRophetic” package to predict responsiveness to therapeutic drugs based on half maximal inhibitory dose (IC50) for each ccRCC sample.

### 2.10 Statistic analysis

R software (version 4.1.3 & 4.2.1), IBM SPSS Statistics (version 26), and GraphPad Prism (version 9) were used to conduct the statistical analyses. The assessment and comparison of survival times was done using Kaplan-Meier (K-M) survival curves. To determine whether there was a correlation between the variables, Spearman or Pearson correlation analysis was used. The Wilcoxon test was used to continuous variables. For all statistical methods, a difference was deemed significant if *p* < 0.05.

## 3 Results

### 3.1 Construction of a 7-chemokine-genes signature for predicting prognosis of ccRCC

**FIGURE 1:**
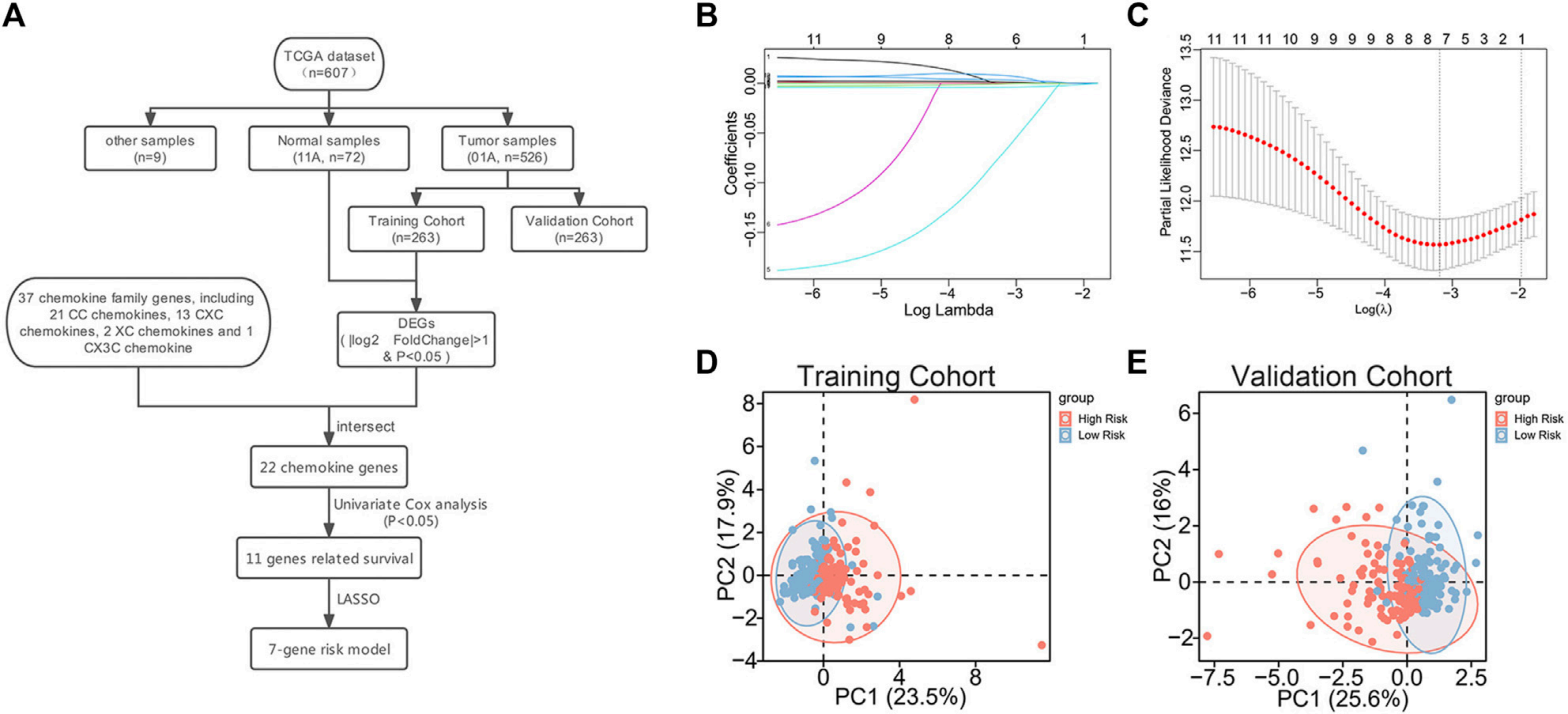
Construction of 7-chemokine-genes prognostic signature. **(A)** Flow chart of model construction. **(B)** Cross-validation was performed to optimize the parameter selection of the LASSO regression model. **(C)** Distribution of lasso coefficients of 11 prognosis-related chemokine genes. **(D, E)** Correlation between chemokine genes signature and profiles of seven chemokine genes’ expression in the training cohort and validation cohort.

A total of 37 chemokines were used in this work, including 21 CC-chemokines, 13 CXC-chemokines, 2 XC-chemokines, and 1 CX3C-chemokine (Supplementary Table S2). We constructed a signature of the 7-chemokine-genes (Figure 1A). Firstly, the TCGA database’s ccRCC tumor samples were separated into a training cohort and a validation cohort at random. Then, the training cohort samples were subjected to differential analysis with normal samples to obtain DEGs (| $\log_{2}$ FoldChange|>1 & *p* < 0.05), which were intersected with the selected 37 chemokine genes to gain 22 chemokine genes. Next, 11 chemokines related to prognosis were discovered by a univariate Cox regression analysis (Supplementary Table S3). Subsequently, we executed the LASSO algorithm and identified 7 chemokines (Figures 1B,C). Finally, the expression values of the seven candidate chemokine genes and their corresponding correlation coefficients were used to construct a prognostic index with the following equation:

$$RiskScore=\sum i=Coef\left(i\right)\times Expr\left(i\right)$$

In addition, to further investigate the association between the signature and the expression profiles of the 7 potential genes, we used principal component analysis (PCA). The results showed a significant association between riskscore and the expression profiles of the seven potential genes (Figures 1D, E).

### 3.2 Association of clinicopathological features of ccRCC with gene signature

**FIGURE 2:**
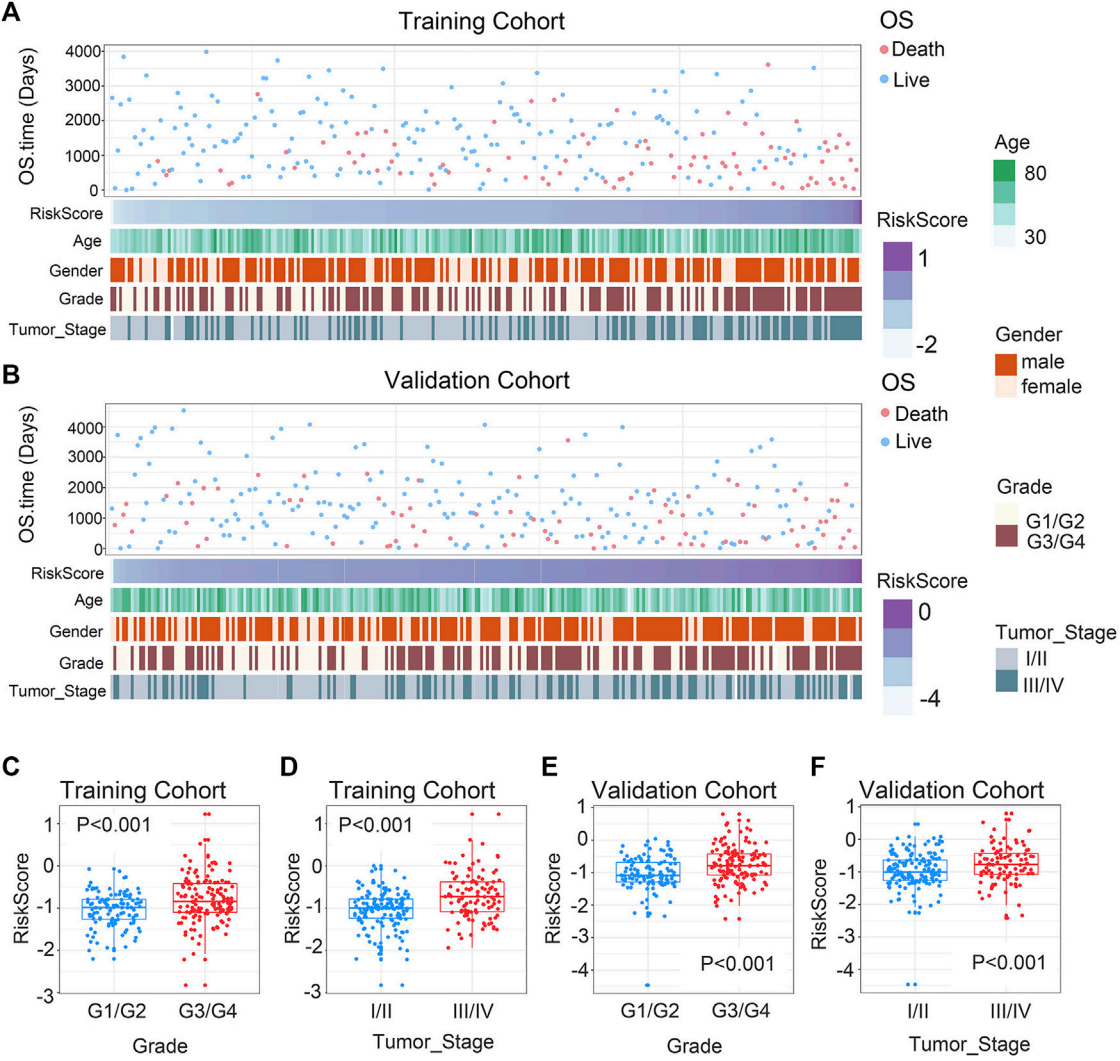
Association between chemokine-based gene signature and clinical features in ccRCC. **(A)** Correlation of riskscore and clinicopathological characteristics of patients in the training cohort. **(B)** Correlation of riskscore and clinicopathological characteristics of patients in the validation cohort. **(C, E)** In the training and validation cohorts, riskscore considerably rise at higher-grade ccRCC. **(D, F)** In the training and validation cohorts, riskscore considerably rise at higher-stage ccRCC. The significance of the difference was tested with Wilcoxon test.

In order to ascertain the clinicopathological implications of this gene signature, we analyzed the relationship between riskscore and clinicopathological information, such as age, gender, histological grade, and tumor stage. Patients with different riskscore exhibited different clinical and pathological features. Histological grade and tumor stage showed an uneven distribution as the riskscore increased in either the training and validation cohorts (Figures 2A, B). The various groups of these samples underwent comparison analysis. Riskscore was higher in high-grade ccRCC in the training cohort (Figure 2C). In addition, stage III/IV tumor samples showed higher riskscore (Figure 2D). The validation cohort verified the aforementioned findings (Figures 2E, F). Overall, these findings collectively imply that the signature and clinicopathological traits are tightly connected.

### 3.3 Prognostic value of the 7-chemokine-genes signature

**FIGURE 3:**
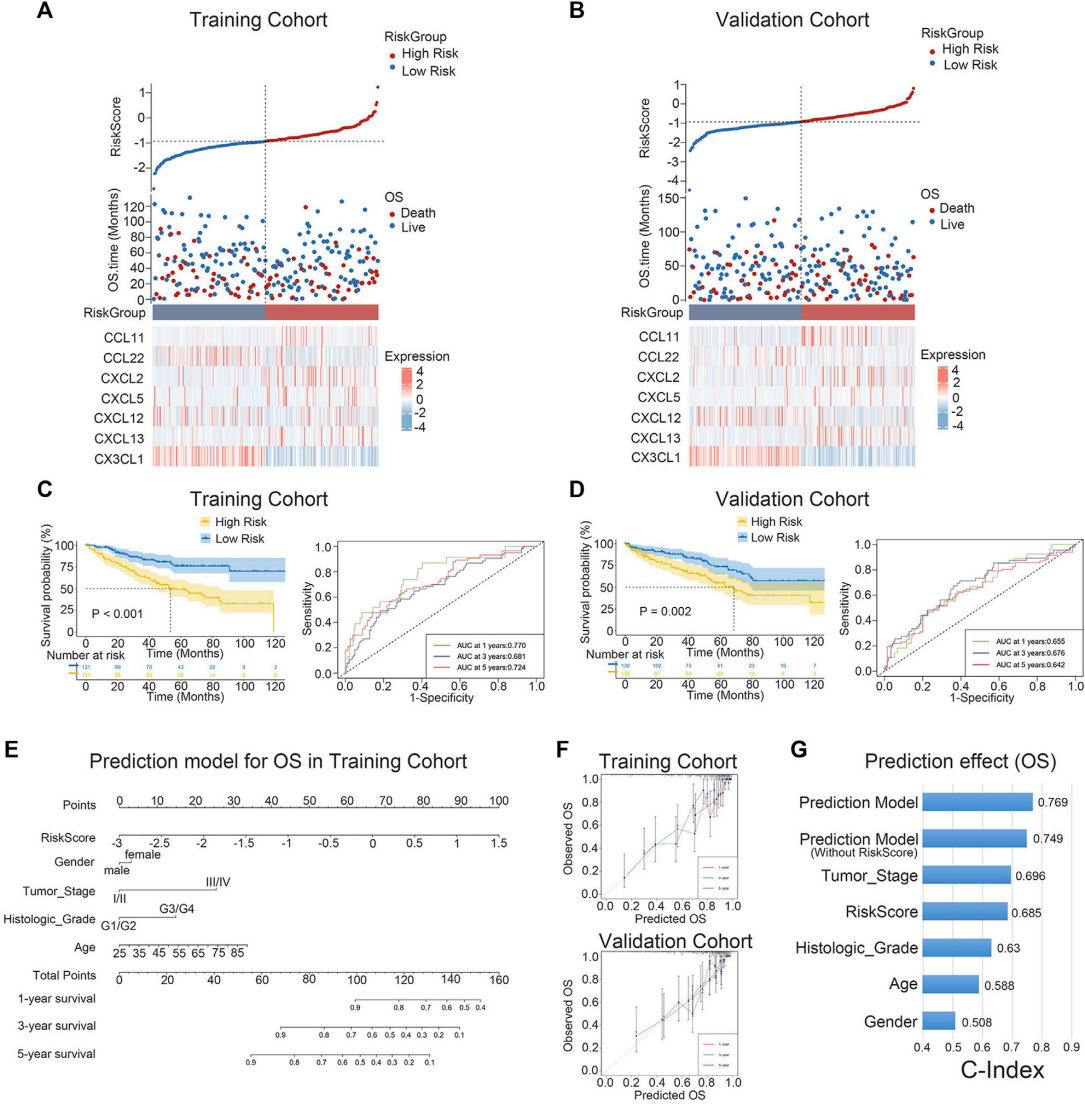
K-M survival analysis and nomogram survival prediction. **(A, B)** Distribution of riskscore, survival status and survival time in ccRCC patients, and heat map of 7 chemokine genes. **(C, D)** K-M survival curves for OS and ROC curves for 1-, 3-, and 5-year survival rates. **(E)** Nomogram prediction combining clinicopathological features and riskscore. **(F)** Predicted and observed 1-year, 3-year and 5-year survival in calibration plots for training and validation cohorts. **(G)** The C-index is used to visualize the predictive effect of predictive model, riskscore, predictive model without riskscore, and clinicopathological factors.

The expression levels of the seven chemokine genes and patient survival times were ordered by riskscore values in order to further evaluate the relationship between signature and overall survival time of patients. All patients were classified into high-risk and low-risk patient groups based on the median riskscore values. (Figures 3A, B). The results of the survival analysis showed that patients with low risk had a significantly better prognosis than those with high risk (Figure 3C). The validation cohort provided strong confirmation of the aforementioned findings (Figure 3D). Notably, for patients with ccRCC in both cohorts, riskscore is shown to be an independent prognostic factor of overall survival times. (Supplementary Tables S4, S5). Furthermore, an individualized prediction model was created to aid in the clinical use of prognostic prediction models. Age, gender, histological grade, tumor stage, and riskscore were included as independent predictors in the construction of the OS prediction model. The results showed that the prediction model may be used to assess the likelihood of 1-, 3-, and 5-year overall survival times in patients with ccRCC. (Figure 3E). Notably, the nomogram and calibration curves actually observed results in the training and validation cohorts are satisfactory, showing excellent prediction accuracy (Figure 3F). This nomogram model has a C-index of 0.769, which is better compared to any other prediction model (Figure 3G). The above results are verified in the validation cohort (Supplementary Figures S1A, B). Collectively, the signature can well predict the prognosis of ccRCC and is expected to translate into clinical applications.

### 3.4 Biological function and signaling pathways analysis

**FIGURE 4:**
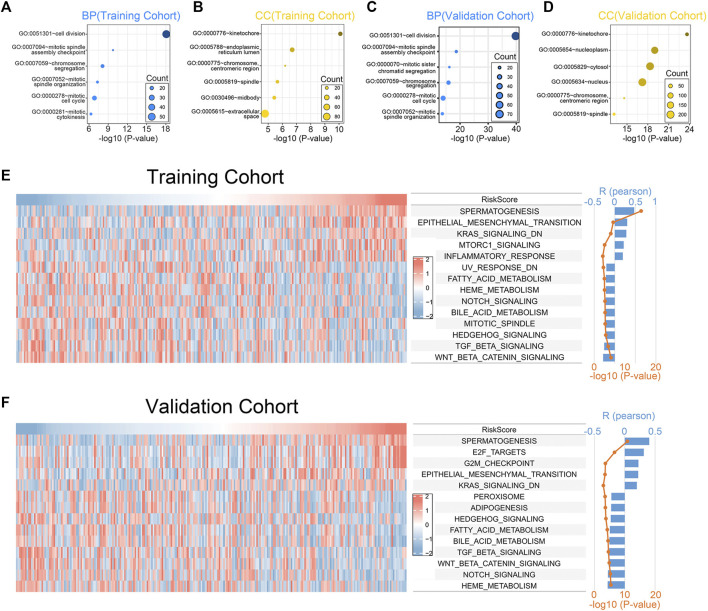
Biological functions associated with the 7-chemokine-genes signature. **(A–D)** The biological processes (BP) and cell components (CC) that are enriched by genes that are positively correlated with riskscore. **(E, F)** Correlation of riskscore with the HALLMARK gene set. The heat map shows the enrichment scores of the HALLMARK for each patient. Bar and line plots show R- and *p*-values for correlation analysis.

To investigate the biological processes and the pathways related to the 7 chemokine genes signature, we used GO and GSVA analyses. First, we sought genes positively associated with riskscore (Pearson correlation, R > 0.3 & *p* < 0.05), finding 657 and 466 genes in the training and validation cohorts, respectively. Then, GO analysis revealed that genes with positively associations were mainly associated with mitosis (Figures 4A–D). In addition, the hallmark analysis also showed that riskscore were positively correlated with EMT and KRAS signaling pathways, but negatively correlated with TGF-β and WNT signaling pathways (Figure 4E). The validation cohort verified the aforementioned findings (Figure 4F). This suggests that our signature can also predict the malignant course of ccRCC.

### 3.5 Comparison of somatic mutations and TMB characteristics

**FIGURE 5:**
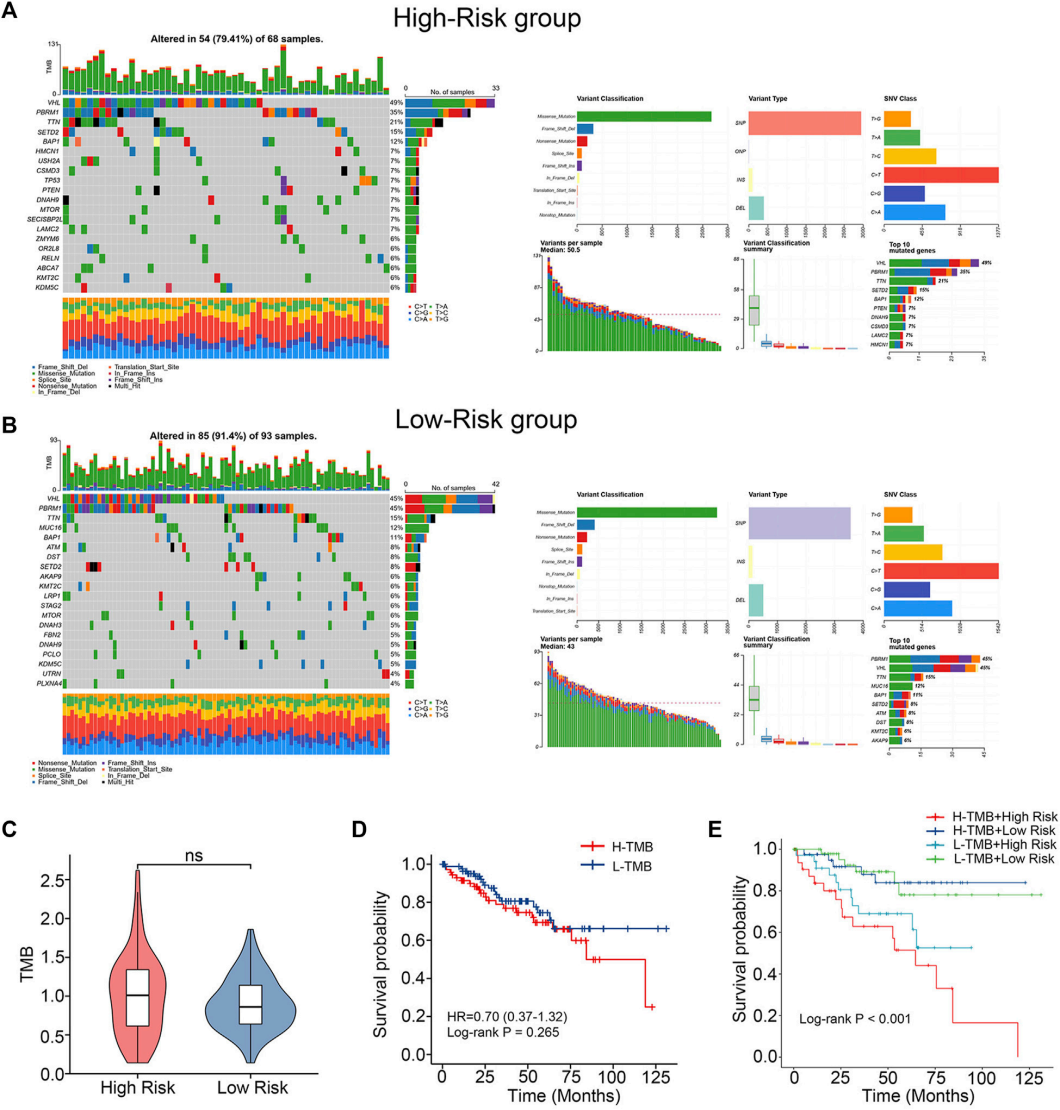
Differences in mutations between high- and low-risk groups. **(A, B)** Somatic mutation waterfall plots in the training cohort. **(C)** TMB difference between low-risk and high-risk group. (Wilcoxon test). **(D)** K-M survival curves comparing the groups with high and low TMB levels. **(E)** Intergroup K-M survival curves for the four groups.

In order to compare the differences in gene mutations between the high-risk and low-risk groups, data on single nucleotide variations were gathered from TCGA. VHL (49%), PBRM1 (35%), TTN (21%), SETD2 (15%) and BAP1 (12%) were the top 5 genes with the highest frequency of mutations in the training cohort’s high-risk group (Figure 5A). In contrast, VHL (45%), PBRM1 (45%), TTN (15%), MUC16 (12%) and BAP1 (11%) were the top 5 genes with the highest frequency of mutations in the training cohort’s low-risk group (Figure 5B). The TMB of the two groups were also compared and no significant differences were found (Figure 5C). No difference in survival time existed between the groups with high and low TMB. (Figure 5D). After merging our models, the high-risk + high-TMB group’s prognosis was noticeably poorer than the low-risk + low-TMB group’s. (Figure 5E). This indicates that our chemokine gene predictive signature combined with TMB can more accurately predict the prognosis of patients.

### 3.6 Single-cell analysis

**FIGURE 6:**
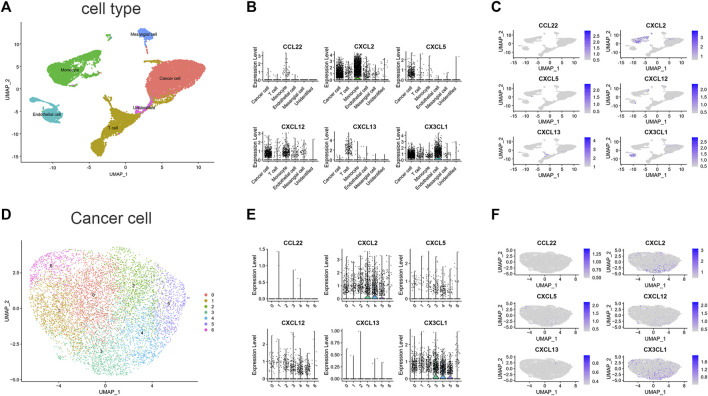
scRNA-seq data analysis in GSE152938. **(A)** Cell-type annotation of clusters. **(B)** Signature genes expression levels in several cell subtypes. **(C)** UMAP shows expression of signature genes in all cell subtypes. **(D)** Subtypes of Cancer cells. **(E)** Signature genes expression levels in different subtypes of cancer cells. **(F)** UMAP shows expression of signature genes in subtypes of cancer cells. (CCL11 is unavailable).

To estimate the TME in patients with ccRCC, we performed scRNA-seq analysis. We first collected single-cell sequencing data from 2 ccRCC patients in the GSE152938 dataset. The dataset’s overall picture was plotted (Supplementary Figures S2A, B). After quality control, quality control visualization and removal of samples with gene expression less than 200 and mitochondrial gene proportion greater than 20%, a total of 22,623 genes and 18,032 cells were preserved. Variable features were set to 2000, and the top 10 genes of the 2000 highly variable features were plotted (Supplementary Figure S2C). Subsequently, PCA was conducted out to show the genes included in the 12 PCs in the PCA (Supplementary Figure S2D). Dimensionality reduction analysis was performed using the “UMAP” method. Cells were clustered into 19 clusters (Supplementary Figure S2E). According to the expression of marker genes (Supplementary Figures S2F, G), we identified five different cell clusters and one unidentified cell cluster (Figure 6A), namely, T cell, Endothelial cell, Mesangial cell, Cancer cell, and Monocyte. The signature genes CXCL2, CXCL12 and CX3CL1 were mainly expressed in cancer cells and monocytes (Figures 6B,C). In addition, we further downscaled the cancer cell subtypes using the “UMAP” method, and the cells were clustered into 7 clusters (Figure 6D), and we investigated the distribution of signature genes across clusters (Figures 6E, F). This suggests that genes in our signature have an impact on the physiological processes of cancer cells, especially the CXCL2 and CX3CL1 genes.

### 3.7 Immune checkpoints and immune cell infiltration associated with gene signatures

**FIGURE 7:**
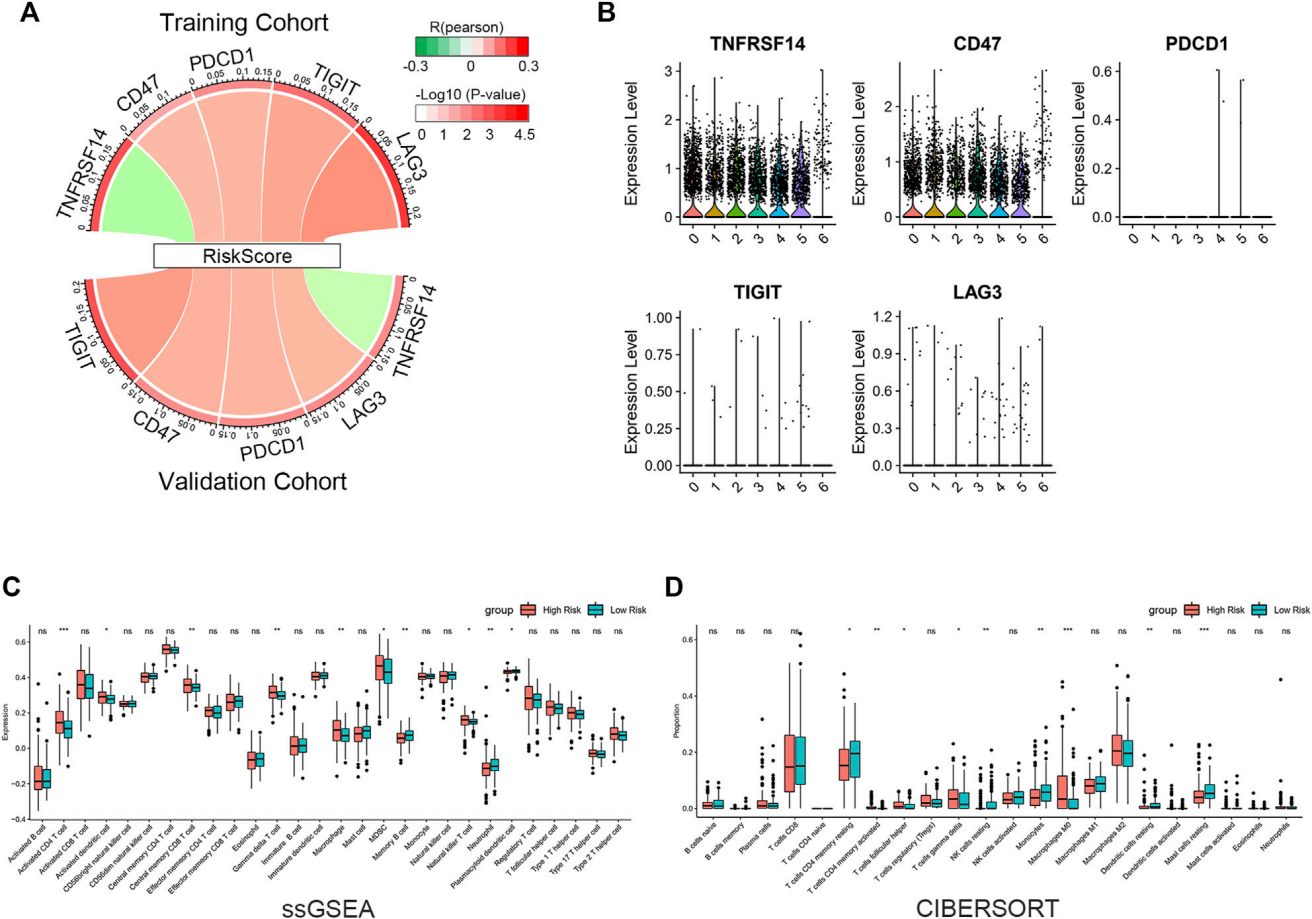
Immune infiltration reflected by gene signature. **(A)** Correlation between riskscore and inhibitory ICs. The R-value is shown by the band’s width. The *p*-value is indicated by the band’s color. The correlation was examined using Pearson correlation analysis. **(B)** Expression levels of inhibitory ICs in various subtypes of cancer cells. **(C)** Comparison of 28 immune cell enrichment scores. **(D)** Comparison of the difference in the abundance of immune infiltrating cells by the CIBERSORT algorithm. The significance of the difference was tested with Wilcoxon test. *p < 0.05, **p < 0.01, and ***p < 0.001.

We evaluated the correlation between riskscore and known suppressive ICs. The findings revealed that the riskscore was positively correlated with CD47, PDCD1, TIGIT, LAG3 and negatively correlated with TNFRSF14 (Figure 7A). scRNA-seq analysis indicated that TNFRSF14 and CD47 were highly expressed in cancer cells (Figure 7B), suggesting that CD47 may be better therapeutic targets. Previous studies have shown that both inflammatory response and TME are essential for the development of tumors. Therefore, we further examined the association between this signature and TME. Using the ssGSEA approach, we compared the enrichment scores of 28 different immune cell types. The analysis revealed a higher enrichment score of activated CD4 T cells, activated dendritic cells, central memory CD8 T cells, gamma delta (γδ) T cells, macrophages, myeloid-derived suppressor cells (MDSC) and natural killer T cells in the high-risk group compared to the low-risk group. In contrast, higher enrichment scores of memory B cells, neutrophils, and plasmacytoid dendritic cells were found in the low-risk group. (Figure 7C). Subsequently, we analyzed the proportion of 22 immune infiltrating cells in the tumor microenvironment using the CIBERSORT method. Our findings reveal that the abundance of monocyte was elevated in the low-risk group and negatively correlated with the riskscore. In contrast, the abundance of γδ T cell and M0 macrophage were positively correlated with the riskscore (Figure 7D). The study imply that the signature may partially reflect the tumor immunological microenvironment.

### 3.8 Identification of potential therapeutic agents

**FIGURE 8:**
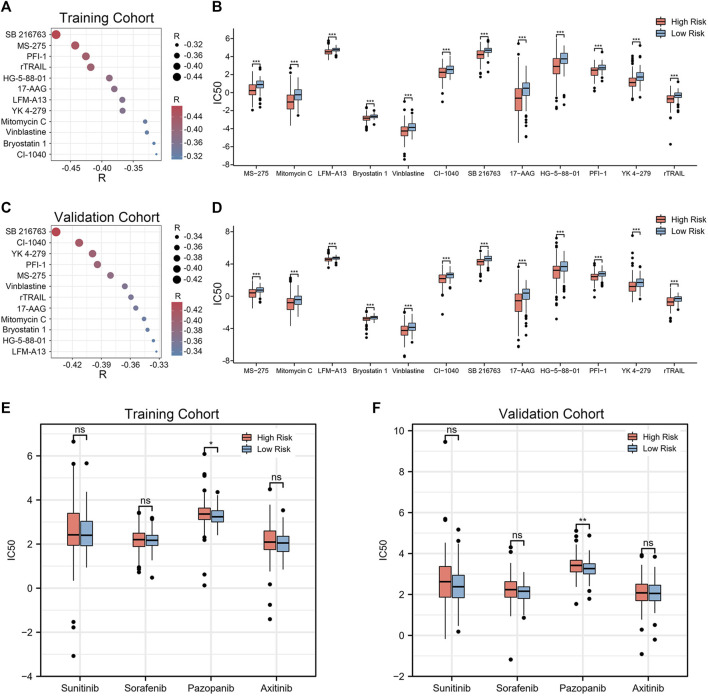
Twelve drug agents were identified as well as four VEGFR inhibitors for analysis. **(A, C)** Correlation between riskscore and the IC50 estimates for the 12 agents. The correlation was examined using Spearman correlation analysis. **(B, D)** Differences in the estimated IC50 for 12 agents between the high- and low-risk groups. **(E, F)** Comparison of IC50 estimates of four VEGFR targeted drugs (sunitinib, sorafenib, pazopanib and axitinib). The significance of the difference was tested with Wilcoxon test. *p < 0.05, **p < 0.01, and ***p < 0.001.

Given that chemotherapy remains a common adjuvant therapy in clinical practice, we explored drug candidates with higher drug sensitivity in high-risk patients. To assess the therapeutic drug response, we determined the IC50 of each ccRCC sample using the “pRRophetic” algorithm. First, compounds with IC50 estimates negatively correlated with riskscore were chosen (Spearman correlation, R < −0.30 & *p* < 0.05). Crossover results between the training and validation cohorts yielded 12 compounds, including SB 216763, MS-275, PFI-1, rTRAIL, HG-5-88–01, 17-AAG, LFM-A13, YK 4–279, Mitomycin C, Vinblastine, Bryostatin 1, CI-1040 (Figures 8A, C). Among them, SB 216763 acted as an inhibitor of GSK-3 targets in the WNT signaling pathway. Further analysis revealed that the IC50 estimates for each of these compounds were lower in the high-risk group (Figures 8B, D). This means that these drugs are promising therapeutic options for ccRCC patients at high risk. Finally, we evaluated the difference in IC50 of several VEGFR inhibitors (sunitinib, sorafenib, pazopanib, and axitinib) in the two cohorts. In contrast to the low-risk group, the high-risk group had higher pazopanib IC50 values (Figures 8E, F). This indicates that high-risk patients may not be sensitive to pazopanib treatment.

## 4 Discussion

Renal clear cell carcinoma is the most common solid cancer in the kidney. (Capitanio and Montorsi, 2016). The advent of targeted therapies and immune checkpoint inhibitors (ICIs) has transformed the treatment of patients with ccRCC. In addition, many targeted therapeutic agents and ICIs have been applied to treat advanced ccRCC. (Ward and Stadler, 2010; Powles et al., 2020; Rini et al., 2020). However, these treatments inevitably lead to some significant adverse events. Chemokines are key players not just in the immune system, but also in the development, growth, and metastasis of tumors. (Strieter et al., 2004; Zlotnik, 2004; Tsaur et al., 2012). Recently, it has been shown that chemokine-based risk signatures show good predictive power in clinical prognosis and response to immunotherapy in glioma, lung adenocarcinoma and pancreatic adenocarcinoma. (Chen et al., 2021; Huang et al., 2021; Fan et al., 2022). As a result, we investigated thoroughly the chemokine genes in ccRCC. First, we selected seven chemokine genes with prognostic value for study. Additionally, we created a brand-new prognostic signature for ccRCC patients and validated it. We discovered that the TME and the response to immunotherapy were connected with our prognostic signature, which may offer practical leads for predicting the prognosis of patients and selecting drug options for patients on immunotherapy.

In the previous study, expression of both CXCR4 and its ligand CXCL12 in VHL-null 786-O cells, even in the lack of exogenous CXCL12, may promote ccRCC proliferation and metastatic dissemination by stimulating autocrine receptors. (Struckmann et al., 2007). In addition, Jin et al. (2019). Reported that miR-34a-5p/CCL22 axis positively regulates the proliferation and metastasis of renal cell carcinoma (RCC). Also, the CCL22-derived peptide vaccination successfully slowed the progression of tumors *in vivo* and demonstrated good therapeutic efficacy. (Lecoq et al., 2022). In other chemokine genes, Parenchymal polymorphonuclear-MDSC (PMN-MDSC) positively correlates with CXCL5, IL1b, IL8, and Mip-1a, which are able to attract PMN-MDSC into ccRCC parenchyma. (Najjar et al., 2017). Interestingly, CXCL5 expression in non-small cell lung cancer was related to a reduced survival rate. (Kowalczuk et al., 2014). In addition, according to research by Dai, et al. (2021), CXCL13+CD8^+^ T cell infiltration levels within tumors are independent predictors of poor OS and RFS in ccRCC and are related with immune evasion of TME. Moreover, CX3CL1 plays a role in tumor promotion and dissemination in patients with RCC besides CXCL12. (Tsaur et al., 2012). There is a lack of articles discussing the tumorigenic aspects of CCL11 and CXCL2 in ccRCC. However, these chemokines have been found to be associated with tumorigenesis and progression in ovarian and hepatocellular carcinoma. (Nolen and Lokshin, 2010; Xu et al., 2021). The gene signature we developed includes each of the chemokines mentioned above. It also implies that this gene signature might play a key role in identifying patients with advanced ccRCC who have poor prognoses and reflecting TME. Moreover, among the clinicopathologic characteristics of malignancy that strongly correlate with high riskscore are high histologic grade and tumor stage. High-risk patients also have shorter survival times. It is noteworthy that this gene signature also functions as an independednt predictor. The nomogram, which included the riskscore and clinicopathological characteristics, demonstrated good accuracy. The aforesaid results were supported by the validation cohort. These findings suggest that the 7-chemokine gene may someday be applied in the clinic.

Chemokines can not only control the migration and localization of immune effector cells in tissues, but also coordinate the interactions between immune cells to reshape the tumor immune microenvironment. (Sokol and Luster, 2015; Nagarsheth et al., 2017). As a result, when we analyzed immune cell infiltration between the high-risk and low-risk groups in our study, we discovered that the high-risk group had a higher density of immune cell infiltration. The high-risk group also showed an increase in T cell activation and antigen-presenting capacity. According to our findings, the high-risk group had greater enrichment fractions of natural killer T cells, activated CD4 T cells, activated dendritic cells, central memory CD8 T cells, γ δ T cells, macrophages, and MDSC. One study reported that MDSC may protect cancer from the patient’s immune system. (Tesi, 2019). These might help to partially explain why the prognosis is worse for patients in the high-risk group. Furthermore, scRNA-seq analysis revealed that CD47 was substantially expressed in tumor cells, and we discovered that riskscore were positively linked with CD47, PDCD1, TIGIT, and LAG3. This shows that the high-risk group may benefit more therapeutically from targeting CD47. The therapeutic potential of CD47 has also been demonstrated in earlier research. (Liu et al., 2015; Logtenberg et al., 2020). Collectively, these results suggest that our 7-chemokine-genes signature may serve as an indicator of the tumor’s immune infiltration status and a promising therapeutic target for immunotherapy of ccRCC patients.

Finally, we obtained 12 drugs that are expected to be therapeutic agents for patients at high risk of ccRCC. Through Genomics of Drug Sensitivity in Cancer (GDSC) and ClinicalTrials.gov (https://clinicaltrials.gov/ct2/home), we obtained the details of these drug candidates (Supplementary Table S6). These drugs act on signaling pathways such as WNT, MAPK, and Apoptosis regulation. Wnt signaling acts as a targeted growth factor to induce cell proliferation and holds promise as a real therapy. (Nusse and Clevers, 2017). Among them, MS-275, 17-AAG, Mitomycin C, Vinblastine, Bryostatin 1, and CI-1040 are already in clinical trials. However, further trials are needed to validate them. Furthermore, we compared four VEGFR inhibitors, sunitinib, sorafenib, pazopanib and axitinib, and showed that the IC50 estimates for pazopanib were elevated in the high-risk group compared to the low-risk group. This indicates that patients in the high-risk group had less responsiveness to the medication pazopanib. In order to prevent overtreatment or adverse effects in non-responders, clinicians can utilize this signature as a predictor of the sensitivity of chemotherapeutic and targeted medicines prior cancer treatment.

However, there are still some limitations of this study that need to be resolved. First off, all of the cohorts used in our analysis were obtained from the TCGA database, and external cohorts are required to confirm the findings. Although the use of an immunotherapy cohort would provide additional insights, our dataset sample size is less than fifty and these data will be presented in our further studies. Second, additional research into the molecular mechanisms needs to be conducted in subsequent studies.

In summary, we established and validated a genetic signature for ccRCC patient prognosis and explored the function of chemokine-related genes in patients with ccRCC. To predict patient OS, a predictive nomogram was created by incorporating factors such as age, sex, tumor stage, histological grade, and riskscore. Also noteworthy, we also developed 12 drug candidates. In addition, the signature can be utilized by clinicians to forecast patient receptivity to targeted and immunochemotherapy treatments.

## Data Availability

The original contributions presented in the study are included in the article/Supplementary Material, further inquiries can be directed to the corresponding authors.
