# Supplementary material for: A novel 7-chemokine-genes predictive signature for prognosis and therapeutic response in renal clear cell carcinoma
Source: Front Pharmacol. 2023 Mar 20;14:1120562. doi: 10.3389/fphar.2023.1120562 (PMC10067584; doi:10.3389/fphar.2023.1120562)
Supplement: Supplementary file 1 [file DataSheet1.PDF]

1.Supplementary Tables

Supplement Table 1. Clinical characteristics of samples

	Training Cohort (N=263)	Validation Cohort (N=263)	All Tumor Samples (N=526)
**Gender**			
female	97 (36.9%)	87 (33.1%)	184 (35.0%)
male	166 (63.1%)	176 (66.9%)	342 (65.0%)
**Age**			
Mean (SD)	60.8 (11.6)	60.4 (12.6)	60.6 (12.1)
Median [Min, Max]	61.0 [29.0, 88.0]	61.0 [26.0, 90.0]	61.0 [26.0, 90.0]
**Histologic Grade**			
G1-G2	119 (45.2%)	119 (45.2%)	238 (45.2%)
G3-G4	141 (53.6%)	139 (52.9%)	280 (53.2%)
GX	2 (0.8%)	3 (1.1%)	5 (1.0%)
not reported	1 (0.4%)	2 (0.8%)	3 (0.6%)
**Pathologic T**			
T1-T2	166 (63.1%)	171 (65.0%)	337 (64.1%)
T3-T4	97 (36.9%)	92 (35.0%)	189 (35.9%)
**Pathologic M**			
M0	203 (77.2%)	213 (81.0%)	416 (79.1%)
M1	43 (16.3%)	35 (13.3%)	78 (14.8%)
MX	16 (6.1%)	14 (5.3%)	30 (5.7%)
not reported	1 (0.4%)	1 (0.4%)	2 (0.4%)
**Pathologic N**			
N0	118 (44.9%)	121 (46.0%)	239 (45.4%)
N1	9 (3.4%)	7 (2.7%)	16 (3.0%)
NX	136 (51.7%)	135 (51.3%)	271 (51.5%)
**Tumor Stage**			
I-II	156 (59.3%)	163 (62.0%)	319 (60.6%)
III-IV	106 (40.3%)	98 (37.3%)	204 (38.8%)
not reported	1 (0.4%)	2 (0.8%)	3 (0.6%)

Supplement Table 2. The details of chemokines

Chemokines	Synonym(s)	Ligand Location	Chemokine Receptor(s)	Receptor Location
**CC Chemokines**				
CCL1	I-309; TCA3	17q11.2	CCR8	3p22
CCL2	MCP-1, MCAF	17q11.2-q12	CCR2	3p21
CCL3	MIP-1α, LD78α	17q11-q21	CCR1, CCR5	3p21
CCL4	MIP-1β, LAG-1, ACT-2	17q11-q23	CCR5	3p21
CCL5	RANTES	17q11.2-q12	CCR1, CCR3, CCR5	3p21
CCL7	MCP-3	17q11.2-q12	CCR2, CCR3	3p21
CCL8	MCP-2	17q11.2	CCR1, CCR3, CCR5	3p21
CCL11	Eotaxin-1	17q21.1-q21.2	CCR3	3p21
CCL13	MCP-4	17q11.2	CCR2, CCR3	3p21
CCL14	HCC-1	17q11.2	CCR1	3p21
CCL15	HCC-2, MIP-5	17q12	CCR1, CCR3	3p21
CCL16	HCC-4, NCC-4, LEC	17q12	CCR1, CCR2, CCR5	3p21
CCL17	TARC	16q13	CCR4	3p24
CCL18	DC-CK1, PARC, MIP-4	17q11.2	CCR8	3p22
CCL19	MIP-3β, ELC, exodus-3	9p13	CCR7	17q12-q21.1
CCL20	MIP-3α, LARC, exodus-1	2q33-q37	CCR6	6q27
CCL21	6Ckine, SLC, exodus-2	9p13	CCR7	17q12-q21.2
CCL22	MDC, STCP-1	16q13	CCR4	3p22
CCL25	TECK, MIP-4a	19p13.2	CCR9	3p21
CCL26	eotaxin-3	7q11.2	CCR3	3p21
CCL28	SCYA28, MEC, CCK1	5p12	CCR3, CCR10	3p21
**CXC Chemokines**				
CXCL1	SCYB1, GRO-α	4q21	CXCR1, CXCR2	2q35
CXCL2	SCYB2, GRO-β	4q21	CXCR2	2q35
CXCL3	SCYB3, GRO-γ, MIP-2β	4q21	CXCR2	2q35
CXCL5	ENA-78$^+$	4q13-q21	CXCR2	2q35
CXCL6	GCP-2$^+$	4q21	CXCR1, CXCR2	2q35
CXCL8	IL-8	4q13-q21	CXCR1, CXCR2	2q35
CXCL9	Mig	4q21	CXCR3	Xq13
CXCL10	IP-10	4q21	CXCR3	Xq13
CXCL11	I-TAC	4q21.2	CXCR3	Xq13
CXCL12	SDF-1α/β	10q11.1	CXCR4	2q21
CXCL13	BLC, BCA-1	4q21	CXCR5	11q23.3
CXCL14	BRAK	5q31	?	
CXCL16	SRPSOX, SCYB16	17p13	CXCR6	3p21
**XC Chemokines**				
XCL1	Lymphotactin α, SCM-1 α	1q21-q25	XCR1	3p21
XCL2	Lymphotactin β, SCM-1 β	1q23	XCR1	3p21
**CX3C Chemokines**				
CX3CL1	Fractalkine	16q13	CX3CR1	3p21

Supplement Table 3. Univariate analysis of chemokines in TCGA Training Cohort

Gene	HR[1]	95% CI[2]		P value
		Low	High	
XCL1	1.04	1.00	1.08	0.044661927
XCL2	1.03	1.01	1.05	0.002264846
CCL1	1.12	0.77	1.61	0.56409413
CCL3	1.00	0.99	1.01	0.885384006
CCL4	1.00	1.00	1.01	0.306535444
CCL5	1.00	1.00	1.00	0.02518071
CCL11	1.03	1.01	1.05	0.001017842
CCL17	1.00	0.97	1.04	0.84588939
CCL18	1.00	1.00	1.00	0.302459778
CCL20	1.00	1.00	1.00	0.232243276
CCL22	0.88	0.80	0.97	0.011549435
CCL25	1.22	1.07	1.40	0.002618371
CCL28	1.00	0.99	1.01	0.93124417
CXCL1	1.00	1.00	1.00	0.141351485
CXCL2	1.00	1.00	1.00	0.017830368
CXCL5	1.00	1.00	1.00	0.009885961
CXCL9	1.00	1.00	1.00	0.985420356
CXCL10	1.00	1.00	1.00	0.375209951
CXCL11	1.00	1.00	1.01	0.513142697
CXCL12	1.00	0.99	1.00	0.047393954
CXCL13	1.00	1.00	1.01	4.57E-05
CX3CL1	0.99	0.99	1.00	7.87E-07

[1] HR = Hazard Ratio, [2]CI = Confidence Interval

Supplement Table 4. Univariate and multivariate analysis of clinical prognostic factors in TCGA Training Cohort

Variable	Univariate analysis			Multivariate analysis		
	HR[1]	95% CI[2]	P value	HR[1]	95% CI[2]	P value
Age	1.03	1.01-1.05	0.001	1.02	1.00-1.04	0.046
Gender	0.91	0.59-1.41	0.681			
Histologic grade	3.24	1.95-5.39	< 0.001	1.79	1.04-3.10	0.037
Tumor stage	4.60	2.89-7.33	< 0.001	2.91	1.77-4.79	< 0.001
RiskScore	4.13	2.82-6.06	< 0.001	2.47	1.61-3.78	< 0.001

[1] HR = Hazard Ratio, [2]CI = Confidence Interval

Supplement Table 5. Univariate and multivariate analysis of clinical prognostic factors in TCGA Validation Cohort

Variable	Univariate analysis			Multivariate analysis		
	HR[1]	95% CI[2]	P value	HR[1]	95% CI[2]	P value
Age	1.03	1.01-1.05	0.003	1.03	1.01-1.05	0.001
Gender	0.98	0.63-1.55	0.945			
Histologic grade	2.23	1.38-3.58	0.001	1.23	0.73-2.06	0.435
Tumor stage	3.35	2.13-5.25	< 0.001	2.72	1.68-4.41	< 0.001
RiskScore	2.32	1.55-3.46	< 0.001	2.25	1.49-3.41	< 0.001

[1] HR = Hazard Ratio, [2]CI = Confidence Interval

Supplement Table 6. The details of candidate drugs

Name	Synonym	Clinical status	Description	Target pathway
SB 216763	Not Available	NO	GSK-3 inhibitor	WNT signaling
MS-275	Entinostat	Phase III	HDAC inhibitor	Chromatin histone acetylation
PFI-1	Not Available	NO	BET inhibitor	Chromatin other
rTRAIL	Not Available	NO	TRAIL receptor agonist	Apoptosis regulation
HG-5-88-01	Not Available	NO	TAK1, M4K2	Other, kinases
17-AAG	Tanespimycin	Phase III	HSP90 inhibitor	Protein stability and degradation
LFM-A13	DDE-28	NO	BTK inhibitor	Other, kinases
YK 4-279	Not Available	NO	RNA helicase A inhibitor	Other
Mitomycin C	MMC	Phase IV	DNA crosslinker	DNA replication
Vinblastine	Velban	Phase III	Microtubule destabiliser	Mitosis
Bryostatin 1	Not Available	Phase II	PKC inhibitor	Other
CI-1040	PD-18435	Phase II	MEK inhibitor	ERK MAPK signaling

2.Supplementary Figures

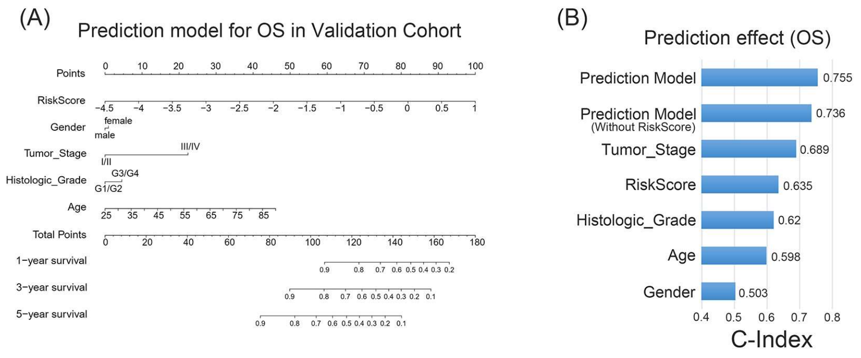

Supplement Figure 1. nomogram survival prediction in validation cohort. (A) Nomogram prediction combining clinicopathological features and riskscore in the validation cohort. (B) The C-index is used to visualize the predictive effect of predictive model, riskscore, predictive model without riskscore, and clinicopathological factors in the validation cohort.

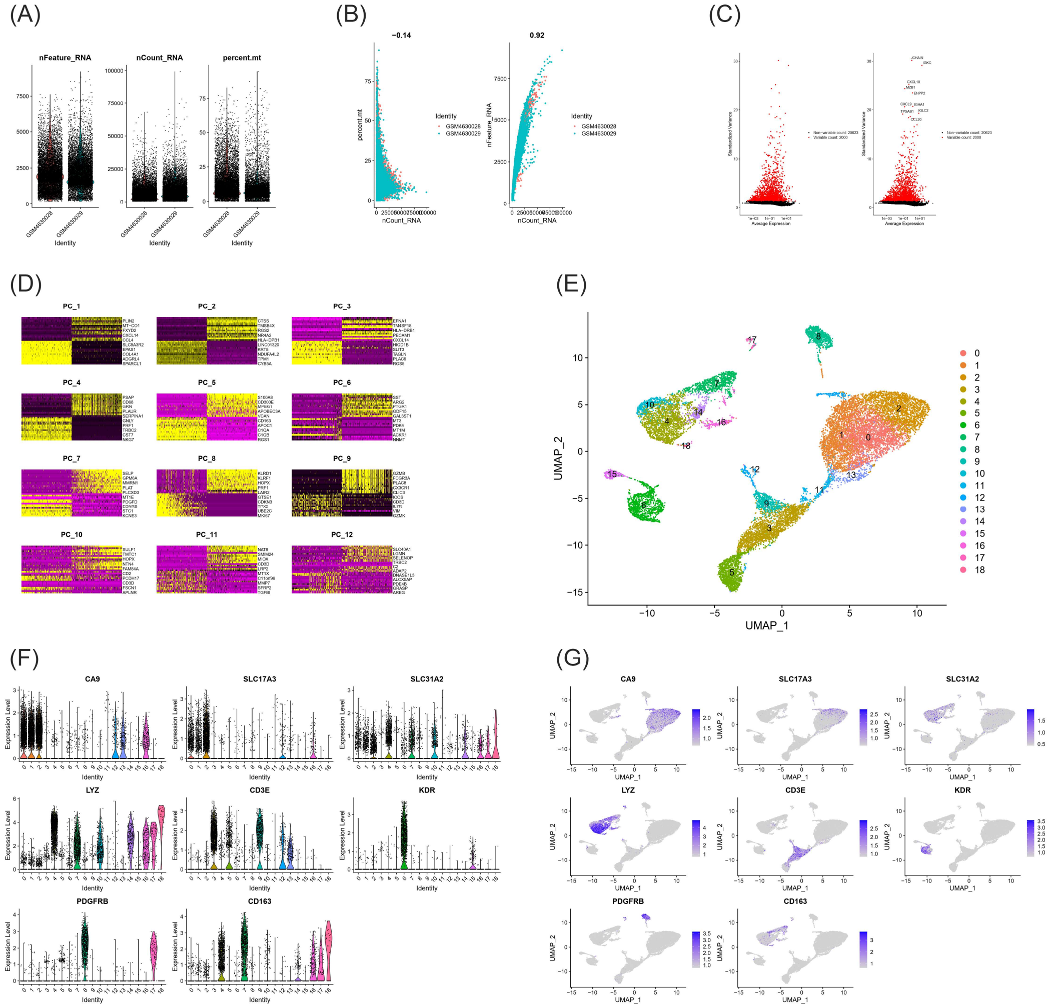

Supplement Figure 2. Quality control, filtering and cell subtype annotation of scRNA-seq data. (A and B) General overview of GSE152938. (C) The top 10 genes of the 2000 highly variable features. (D) Genes contained in the 12 PCs in PCA. (E) 19 clusters. (F and G) Expression of marker genes.
